# Supplementary material for: A reduction in self-reported confidence accompanies the recall of memories distorted by prototypes
Source: Commun Psychol. 2024 Jul 1;2:58. doi: 10.1038/s44271-024-00108-2 (PMC11332036; doi:10.1038/s44271-024-00108-2)
Supplement: Supplementary file 2 — Supplementary Material [file 44271_2024_108_MOESM2_ESM.pdf]

Supplementary Materials

for

A reduction in self-reported confidence accompanies the recall of memories distorted by prototypes

Casper Kerrén, Yiming Zhao & Benjamin Griffiths

Contents:

Supplementary Note 1: Preregistration ………………………………………………….. 2

Supplementary Figure 1 ………………………………………………………….…….. 4

Supplementary Figure 2 ………………………………………………………….…….. 5

Supplementary Table 1 …………………………………………………………….... 6

Supplementary Note 1: Pre-registration

Section	Pre-registered Plan	Deviations
Hypotheses	H1: Participants will cluster their colour bar selections to a greater degree when recalling the colour relative to when perceiving the colour.<br>H2: The degree of clustering will be greater when participants are less confident about the associated colour.<br>H3: Even when highly confident (i.e., selecting 'Sure' during recall), clustering will be greater than what is observed during perception.	N/A
Study type	Experiment - A researcher randomly assigns treatments to study subjects, this includes field or lab experiments. This is also known as an intervention experiment and includes randomized controlled trials.	N/A
Blinding	No blinding is involved in this study.	N/A
Study design	Within-subjects design with 3 variables of interest (condition, clustering, confidence). "Condition" refers to whether participants report the object colour when perceiving the object-colour association or when recalling the object-colour association. "Clustering" refers to a continuous variable that measures the degree to which individual colour bar selections cluster together. "Confidence" refers to a discrete variable that measures recall confidence using a three point scale ("sure", "unsure", "guess")	N/A
Existing Data	Registration prior to creation of data	N/A
Data collection procedures	Data will be collected using Prolific to recruit participants (self-reported fluent English speakers; self-reported age between 18 and 35) and Gorilla to deliver the experiment. Participants will be paid £5 to take part in the study.	N/A
Sample size	27 participants will be recruited. If a given participant produces fewer than 10 "sure", "unsure", and "guess" responses, they will be replaced so that the final sample returns to the final value of 27 participants.	N/A
Sample size rationale	Piloting suggests a very large effect size (d ~ 0.9 to 1.9). A power analysis suggests that a one-tailed design with a significance level of 0.05, statistical power of 0.99, and an effect size of 0.8, would require 27 participants.	N/A
Manipulated variables	"Condition" (i.e., whether participants report the colour during perception or during retrieval) will be manipulated. An equal number of perception and retrieval trials will be completed.	N/A
Measured variables	"Reported colour" will be recorded (for both perception and retrieval trials) as the selected position on the colour bar. "Confidence" will be recorded on retrieval trials as "sure", "unsure" or "guess".	N/A
Indices	Clustering will be computed by first applying an iterative k-means clustering algorithm to the data, where k takes all integer values between 1 and 10 (inclusive). "Clustering error" will then be computed as the mean distance between every "reported colour" and their nearest cluster. centroid. This produces an exponentially declining curve, where the area under curve (AUC) will be used as a measure of "clustering". A smaller AUC indicates a greater tendency to cluster.<br>As the k-means algorithm may be influenced by the number of data points (which may vary based on confidence ratings), "clustering" will also be computed for "optimal" performance on the same subset of trials. "Optimal" performance will set " reported colour" as the	Clustering was replaced with “prototypicality” (as reported in the main text). This change made as the pre-registered clustering approach does not account for the difference between an individual item and its nearest cluster.

	target colour (i.e., as if the participant performed the task flawlessly). The observed clustering will then be standardised against "optimal" clustering by simply subtracting the "optimal" clustering AUC.	
Statistical models	Clustering observed during perception will be contrasted against clustering observed during retrieval using a repeated-measures one-tailed t-test, with the expectation that clustering will be greater during retrieval.<br>A 2x3 repeated-measures ANOVA (factors: condition, confidence) will explore the possibility of an interaction where clustering is greater during retrieval specifically when confidence is low. Three follow-up t-tests will assess whether clustering is greater during retrieval relative to perception for the three confidence conditions, with the false discovery rate being used to control for multiple comparisons.	In the main analyses, “Unsure” and “Guessed” responses were pooled as many participants would need to be excluded (~42%) if these were to be analysed separately.
Inference criteria	An effect will be considered significant if $p < 0.05$	N/A
Data exclusion	Participants will be excluded if they have fewer than 10 "sure" responses, 10 "unsure" responses or 10 "guess" responses as the k-means algorithm requires more samples than suggested clusters.	This criterion was not applied to the analyses in the main text as prototypicality computes clusters over all conditions, rather than each condition separately, and therefore does not require this constraint.
Missing data	Participants that do not complete the study will excluded. Participants automatically rejected by Prolific or Gorilla will be excluded.	N/A

Pre-registered Analyses

Methods

The sample and data analysed here are the same as those reported in Experiment 1. The analysis approach matches that reported in the "Pre-registered Plan" column above.

Results

Repeated measures t-tests found that clustering at retrieval was significantly greater than at perception ($t_{25} = -6.06$, $p < 0.001$), supporting Hypothesis 1.

A 2x3 repeated-measures ANOVA found a significant main effect of condition (termed "epoch" in the main text) on clustering [$F(1, 25) = 36.71$, $p < 0.001$, $\eta_p^2 = 0.60$]. This ANOVA also revealed a main effect of confidence on clustering [$F(1, 25) = 26. 80$, $p < 0.001$, $\eta_p^2 = 0.52$], and a marginal effect for the interaction between confidence and condition [$F(1, 25) = 2.86$, $p = 0.067$, $\eta_p^2 = 0.10$; however the assumption of sphericity was violated: Mauchly's $W = 0.651$, $p = 0.006$]. The marginal effect provides inconclusive evidence for Hypothesis 2 (see supplementary figure 2).

Post-hoc tests suggest that clustering was greater during retrieval than perception for all confidence ratings (Sure: $t_{25} = 4.64$, $p < 0.001$, Cohen's $d = 0.909$, 95% CI = [0.02, 0.04]; Unsure: $t_{25} = 3.22$, $p = 0.004$, Cohen's $d = 0.632$, 95% CI = [0.03, 0.13]; Guess: $t_{25} = 4.00$, $p < 0.001$, Cohen's $d = 0.784$, 95% CI = [0.05, 0.15])), providing support for Hypothesis 3.

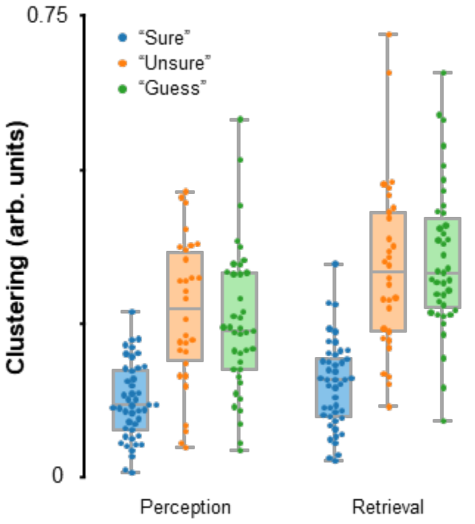

Supplementary Figure 1. Boxplots for data from Experiment 1, depicting clustering given condition (x-axis) and confidence rating (hue) for Experiment 1. The boxplots display the median and interquartile range, with the whiskers capturing the range of the data. Individual dots reflect individual participants.

Discussion

The analyses presented here largely conform to those reported in the main text, though the interaction term is substantially weaker. Speculatively, the suboptimal pre-registered analysis strategy is culpable here. This approach is flawed for several reasons. First, it does not account for the distance between a target and its nearest cluster, which introduces a large source of uncontrolled variance between trials. Second, it computes clusters for each confidence rating separately, which may result in bias when trial numbers differ substantially. While we attempted to control this using a baseline reflecting "optimal" performance for a given number of trials, some residual variance was bound to remain. In the main text, we addressed both concerns by devising the prototypicality measure. For the reasons outlined above, we feel that prototypicality offers substantially greater statistical power to detect clustering in perceptual and recall data. The adoption of prototypicality also meant that we did not have to discard participants for a lack of trials, meaning the sample size could almost double. This additional power undoubtedly further helps in the detection of clustering effects. Critically, while we adapted our analysis of Experiment 1 to account for these issues post-registration, we go on to replicate these effects in a further five experiments, suggesting the results of Experiment 1 reported in the main text are not a statistical fluke introduced by multiple comparisons.

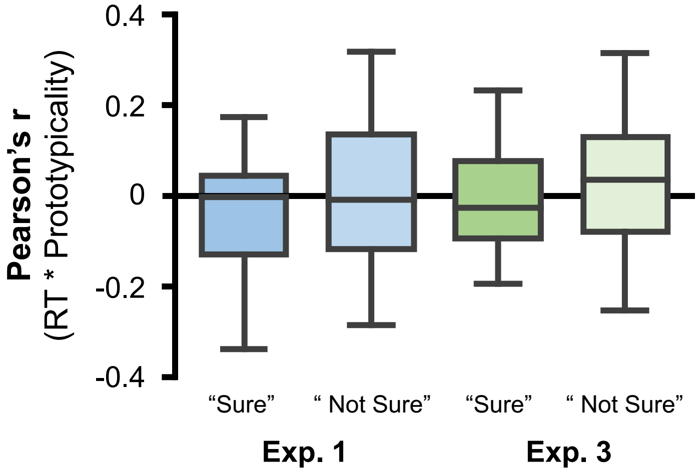

Supplementary Figure 2. To explore a link between reaction time (RT) and prototypicality, we used Pearson's correlation coefficient to correlate response time (when making the colour judgment) with the prototypicality of responses for each participant individually, then used a one-sample t-test to examine whether there was a consistent trend in correlation across participants. In the first instance, we restricted these analyses to Experiments 1 and 3 (the base colour and location experiments) so that we did not need to worry about additional covariates. In Experiment 1, we found no effect for perceptual nor retrieval responses when correlating across all trials (perception: t(44) = 0.22, p = 0.827; retrieval: t(44) = -1.43, p = 0.161). Similar, we found no effect for perceptual or retrieval responses in Experiment 3 (perception: t(33) = -0.93, p =0.361; retrieval: t(33) = 0.25, p = 0.803). The boxplots display the median and interquartile range, with the whiskers capturing the range of the data (Experiment 1: n=45 participants; Experiment 3: n=29 participants).

Supplementary Table 1. Main analyses excluding overshoots and repulsions. No statistical test incurred a change in p-value that crossed the threshold of alpha = 0.05, though numerical changes in F-statistics and p-values nonetheless occurred. Note: some participants were rejected from this analysis as the exclusion of overshoot/repulsion trials meant they did not have any trials in at least one of the analysed conditions.

Exp.	Contrast	F	df	p
1	Confidence	90.93	(1, 44)	< 0.001
	Epoch	119.79	(1, 44)	< 0.001
	Confidence * Epoch	132.80	(1, 44)	< 0.001
2	Confidence	90.94	(1, 32)	< 0.001
	Epoch	293.93	(1, 32)	< 0.001
	Orientation	1.71	(1, 32)	0.200
	Confidence * Epoch	109.45	(1, 32)	< 0.001
	Confidence * Orientation	0.20	(1, 32)	0.660
	Epoch * Orientation	< 0.01	(1, 32)	0.964
	Confidence * Epoch * Orientation	0.42	(1, 32)	0.520
3	Confidence	72.04	(1, 27)	< 0.001
	Epoch	243.83	(1, 27)	< 0.001
	Confidence * Epoch	77.96	(1, 27)	< 0.001
4	Confidence	97.77	(1, 35)	< 0.001
	Epoch	181.83	(1, 35)	< 0.001
	Retrieval Order	2.73	(1, 35)	0.107
	Confidence * Epoch	193.75	(1, 35)	< 0.001
	Confidence * Retrieval Order	0.82	(1, 35)	0.370
	Epoch * Retrieval Order	0.02	(1, 35)	0.887
	Confidence * Epoch * Retrieval Order	0.79	(1, 35)	0.381
5	Confidence	100.43	(1, 38)	< 0.001
	Epoch	66.41	(1, 38)	< 0.001
	Kernel Size	26.31	(1, 38)	< 0.001
	Confidence * Epoch	128.13	(1, 38)	< 0.001
	Confidence * Kernel Size	0.15	(1, 38)	0.698
	Epoch * Kernel Size	28.96	(1, 38)	< 0.001
	Confidence * Epoch * Kernel Size	9.82	(1, 38)	0.003
6	Confidence	6.01	(1, 36)	0.019
	Epoch	0.51	(1, 36)	0.481
	Confidence * Epoch	5.79	(1, 36)	0.021
