# Supplementary material for: A reduction in self-reported confidence accompanies the recall of memories distorted by prototypes
Source: Commun Psychol. 2024 Jul 1;2:58. doi: 10.1038/s44271-024-00108-2 (PMC11332036; doi:10.1038/s44271-024-00108-2)
Supplement: Supplementary file 3 — Reporting Summary [file 44271_2024_108_MOESM3_ESM.pdf]

nature portfolio

Corresponding author(s): Benjamin J. Griffiths

Last updated by author(s): May 16, 2024

Reporting Summary

Nature Portfolio wishes to improve the reproducibility of the work that we publish. This form provides structure for consistency and transparency in reporting. For further information on Nature Portfolio policies, see our Editorial Policies and the Editorial Policy Checklist.

Statistics

For all statistical analyses, confirm that the following items are present in the figure legend, table legend, main text, or Methods section.

n/a	Confirmed	
☐	☒	The exact sample size (*n*) for each experimental group/condition, given as a discrete number and unit of measurement
☐	☒	A statement on whether measurements were taken from distinct samples or whether the same sample was measured repeatedly
☐	☒	The statistical test(s) used AND whether they are one- or two-sided<br>*Only common tests should be described solely by name; describe more complex techniques in the Methods section.*
☒	☐	A description of all covariates tested
☐	☒	A description of any assumptions or corrections, such as tests of normality and adjustment for multiple comparisons
☐	☒	A full description of the statistical parameters including central tendency (e.g. means) or other basic estimates (e.g. regression coefficient) AND variation (e.g. standard deviation) or associated estimates of uncertainty (e.g. confidence intervals)
☐	☒	For null hypothesis testing, the test statistic (e.g. *F*, *t*, *r*) with confidence intervals, effect sizes, degrees of freedom and *P* value noted<br>*Give P values as exact values whenever suitable.*
☒	☐	For Bayesian analysis, information on the choice of priors and Markov chain Monte Carlo settings
☒	☐	For hierarchical and complex designs, identification of the appropriate level for tests and full reporting of outcomes
☐	☒	Estimates of effect sizes (e.g. Cohen's *d*, Pearson's *r*), indicating how they were calculated

Our web collection on statistics for biologists contains articles on many of the points above.

Software and code

Policy information about availability of computer code

Data collection	All data collection was done using Gorilla Experiment Builder platform (www.gorilla.sc). Participants were recruited via Prolific (www.prolific.com) or the university research participation scheme.
Data analysis	All analysis was conducted using custom Python 3 scripts (available at https://osf.io/pmcqh/). Statistical results were cross-checked in JASP 0.18.1.0. Confidence intervals for Cohen's d and partial eta-squared effect sizes were computed using an online tool: https://effect-size-calculator.herokuapp.com/.

For manuscripts utilizing custom algorithms or software that are central to the research but not yet described in published literature, software must be made available to editors and reviewers. We strongly encourage code deposition in a community repository (e.g. GitHub). See the Nature Portfolio guidelines for submitting code & software for further information.

Data

Policy information about availability of data

All manuscripts must include a data availability statement. This statement should provide the following information, where applicable:

- Accession codes, unique identifiers, or web links for publicly available datasets
- A description of any restrictions on data availability
- For clinical datasets or third party data, please ensure that the statement adheres to our policy

The numerical dataset necessary to interpret, replicate and build upon the findings reported here is available in .csv format at https://osf.io/pmcqh/.

Human research participants

Policy information about studies involving human research participants and Sex and Gender in Research.

Reporting on sex and gender	No sex- or gender-based analysis was conducted.
Population characteristics	A total of 218 participants were recruited (~71.3% female; mean age = ~24.0 years [s.d.: ~5.8 years]; for breakdown by experiment, see Table 1).
Recruitment	For Experiments 1 and 3, participants were recruited using Prolific (www.prolific.com) and received financial reimbursement for their time. Participants must have reported being fluent in English, aged between 18 and 35, and residing in the United Kingdom. For Experiments 2, 4, 5, and 6, participants were recruited using the University of Birmingham Research Participation Scheme and received course credit for their participation. All participants reported being aged between 18 and 35, with fluency in English and residence in the United Kingdom being assumed given they had enrolled in the university psychology undergraduate course.
Ethics oversight	University of Birmingham STEM Ethics Committee

Note that full information on the approval of the study protocol must also be provided in the manuscript.

Field-specific reporting

Please select the one below that is the best fit for your research. If you are not sure, read the appropriate sections before making your selection.

☐ Life sciences ☒ Behavioural & social sciences ☐ Ecological, evolutionary & environmental sciences

For a reference copy of the document with all sections, see nature.com/documents/nr-reporting-summary-flat.pdf

Behavioural & social sciences study design

All studies must disclose on these points even when the disclosure is negative.

Study description	The study consists of six online behavioural experiments that use a within-subjects design.
Research sample	A total of 218 participants were recruited (~71.3% female; mean age = ~24.0 years [s.d.: ~5.8 years]; for breakdown by experiment, see Table 1). For Experiments 1 and 3, participants were recruited using Prolific (www.prolific.com) and received financial reimbursement for their time. Participants must have reported being fluent in English, aged between 18 and 35, and residing in the United Kingdom. For Experiments 2, 4, 5 and 6, participants were recruited using the University of Birmingham Research Participation Scheme and received course credit for their participation. All participants reported being aged between 18 and 35, with fluency in English and residence in the United Kingdom being assumed given they had enrolled in the university psychology undergraduate course.
Sampling strategy	The sample size of Experiment 1 was determined based upon sample sizes of similar studies. The remaining experiments used sample sizes that ensured > 95% power based on the data obtained in Experiment 1.
Data collection	All data was collected online using the Gorilla Experiment Builder platform (www.gorilla.sc)
Timing	Data collection began in July 2023 and ended around October 2023. There were momentary gaps in collection between the experiments, but no gaps within an experiment.
Data exclusions	Participants who failed to complete the experiment in 1 hour were automatically discarded by Gorilla Experiment Builder. No other data exclusions took place.
Non-participation	No participants withdrew from the experiment.
Randomization	Participants were not divided into experimental groups

Reporting for specific materials, systems and methods

We require information from authors about some types of materials, experimental systems and methods used in many studies. Here, indicate whether each material, system or method listed is relevant to your study. If you are not sure if a list item applies to your research, read the appropriate section before selecting a response.

Materials & experimental systems

n/a	Involved in the study
☒	☐ Antibodies
☒	☐ Eukaryotic cell lines
☒	☐ Palaeontology and archaeology
☒	☐ Animals and other organisms
☒	☐ Clinical data
☒	☐ Dual use research of concern

Methods

n/a	Involved in the study
☒	☐ ChIP-seq
☒	☐ Flow cytometry
☒	☐ MRI-based neuroimaging
